# A Novel Food-Derived Particle Enhances Sweet and Salty Taste Responses in Mice

**DOI:** 10.3390/nu18010098

**Published:** 2025-12-27

**Authors:** Yuko Kawabata, Junichi Yamazoe, Emiko Imamura, Yuki Nagasato, Yihung Lee, Mami Shinoda, Kirari Koda, Yuki Tomita, Hina Ito, Shingo Takai, Keisuke Sanematsu, Makoto Ogata, Hiroyuki Kono, Noriatsu Shigemura

**Affiliations:** 1 Section of Oral Neuroscience, Graduate School of Dental Science, Kyushu University, 3-1-1 Maidashi, Higashi-ku, Fukuoka 812-8582, Japan; 2 Section of Geriatric Dentistry and Perioperative Medicine in Dentistry, Kyushu University Hospital, 3-1-1 Maidashi, Higashi-ku, Fukuoka 812-8582, Japan; 3 Oral Health/Brain Health/Total Health Research Center, Graduate School of Dental Science, Kyushu University, 3-1-1 Maidashi, Higashi-ku, Fukuoka 812-8582, Japan; 4 Research and Development Center for Five-Sense Devices, Kyushu University, 744 Motooka, Nishi-ku, Fukuoka 819-0395, Japan; 5 Faculty of Food and Agricultural Sciences, Fukushima University, 1 Kanayagawa, Fukushima 960-1296, Japan; 6 Division of Applied Chemistry and Biochemistry, National Institute of Technology, Tomakomai College, Nishikioka 443, Tomakomai 059-1275, Japan

**Keywords:** novel food-derived particle, α-cyclodextrin, xanthan gum, pickering emulsion, taste enhancement, sweet taste, salty taste, sugar and salt reduction, mouse model

## Abstract

Background/Objectives: Reducing the dietary intake of sugar and salt is considered a key strategy for preventing the onset and progression of lifestyle-related diseases. However, these dietary interventions often compromise the taste of foods, which can reduce patient satisfaction. To address this challenge, we focused on novel food-derived particles (NFPs; patent number P7383867) consisting of lipid, α-cyclodextrin, and xanthan gum formulated as an emulsion with excellent retention and diffusion properties. Methods: Here, we investigated the effects of NFPs on the taste responses of mice. Results: In two-bottle preference tests (*n* = 4–6), NFPs enhanced preferences for sweet and salty stimuli in behavioral tests (one-way ANOVA, *p* < 0.05) and increased the responses of the chorda tympani nerve (*n* = 6–8) to sweet and salty stimuli (two-way ANOVA, main treatment effect *p* < 0.05), but had no effect on the responses to sour, bitter, or umami stimuli. Conclusion: These findings suggest that NFPs may enhance peripheral taste responses to sweet and salty flavors, thereby helping maintain the palatability of foods with reduced sugar or salt content. Such modulation may have broad applications in improving the acceptability of therapeutic or restricted diets and supporting both disease management and prevention, including lifestyle-related diseases, kidney disease, and other conditions requiring dietary restriction and may offer translational relevance for human dietary interventions.

## 1. Introduction

In modern public health, the prevention of lifestyle-related diseases such as obesity, type 2 diabetes, dental caries, hypertension, and cardiovascular diseases, as well as the extension of healthy life expectancy, are critical social challenges. Reducing excessive intake of sugar and salt is identified as a core strategy to address these issues [1,2,3,4,5,6]. The World Health Organization (WHO) recommends limiting free sugar intake to less than 10% of total energy intake [7], equivalent to about 50 g per day for an average adult. However, in many regions, the actual intake far exceeds this recommendation. A previous study showed that the consumption of sugar-sweetened beverages among children and adolescents aged 3–19 years in 185 countries increased by 23% from 1990 to 2018, paralleling the increase in the prevalence of obesity in this population [7]. In contrast, a long-term study in the United Kingdom reported that the approximate sugar equivalent in diets decreased by approximately 10% between 2008 and 2019, which was attributed to policy interventions, changes in consumer awareness, and food reformulation. However, people still consume huge amounts of dietary sugar in their food and beverages, and many patients suffer from obesity and diabetes [8,9]. Regarding salt intake, the WHO recommends less than 5 g per day, but the average intake worldwide significantly exceeds this, and salt reduction policies are being implemented [5,10]. It is estimated that 1.65 million deaths from cardiovascular disease in 2010 were attributable to sodium intake exceeding 2.0 g per day [4]. However, foods with reduced sugar or salt are often perceived as “tasteless” or “unpalatable.” This has led to decreased consumer satisfaction, so it is very difficult to maintain a therapeutic diet in daily life [11,12]. In addition, low adherence to dietary therapy in patients with heart failure or myocardial infarction is closely associated with high hospitalization rates. Research has shown that one of the factors influencing adherence to dietary therapies is that eating is a source of pleasure and enjoyment [13,14,15,16,17].

Taste is a key aspect of the enjoyment of eating, and it is a basic physiological function. Essentially, taste is a sensory system that identifies nutrients and harmful substances in food. Taste is detected and identified by the taste buds as sweet, salty, sour, bitter, and umami. Taste buds embedded in the mucous membrane of the tongue are composed of taste cells specific to each taste quality. Various taste substances are detected by taste receptors expressed in taste cells, activating intracellular signaling pathways and ultimately transmitting information to the central nervous system via taste nerves [18,19]. Such peripheral taste mechanisms are regulated by physical and physiological factors in the oral cavity. Recent studies have reported that food-derived components influence taste responses through these mechanisms [18,20,21].

The development of methods to compensate for taste intensity is urgently needed to develop effective dietary therapies. Previous studies have reported that food-derived peptides and polysaccharides can enhance taste. For example, γ-glutamyl peptides have been reported to comprehensively enhance basic tastes such as sweetness, saltiness, and umami, with γ-Glu–Ala (γ-EA) showing particularly strong enhancing power [22,23,24,25,26,27]. However, evidence regarding its stability under various food processing and storage conditions (such as heat or acidic environments) and its effects on sensory characteristics such as texture and aftertaste remains limited [27,28]. Other food-derived taste modulators include high-potency natural sweeteners such as steviol glycosides from *Stevia rebaudiana* and mogrosides from monk fruit (*Siraitia grosvenorii*), both of which are widely used as non-caloric sweeteners and provide high sweetness intensity at low concentration [29,30].

In this study, we focused on novel food-derived particles (NFPs; patent no. P7383867) as candidate materials for physically and chemically regulating taste responses to sweet and salty stimuli. The NFPs were composed of lipid, α-cyclodextrin (α-CD), and xanthan gum, formulated as an emulsion with excellent substance retention, diffusion, and stability properties. α-Cyclodextrin is a cyclic oligosaccharide composed of six α-1,4-linked glucose units. Its hydrophilic outer surface and hydrophobic central cavity enable it to form inclusion complexes with hydrophobic molecules [31]. These properties enable α-CD to encapsulate lipophilic flavor compounds and drugs, thereby improving their solubility and stability. Additionally, α-CD exhibits emulsifying properties, stabilizing water-in-oil emulsions and regulating the spatial distribution and release kinetics of lipophilic drugs in the gastrointestinal tract [32,33]. Xanthan gum is a high-molecular-weight polysaccharide that is widely used as a thickener and stabilizer in food applications. It alters the viscosity of emulsions [34]. The combination of α-CD, oil, and xanthan gum is an example of a Pickering emulsion, i.e., an emulsion with a complex, stable structure with improved retention and diffusion properties of incorporated substances [35,36]. It is expected that these properties of the emulsion will hold true for a wide range of incorporated substances, in this case, oral taste substances. Reports suggesting that viscosity and fluid dynamics within the porous structure of the tongue surface influence the transport and perception of taste substances may support this hypothesis [37]. Although these mechanisms are plausible, they remain hypothetical and were not directly tested in this study.

In this study, we evaluated the effects of the physicochemical properties of NFPs on taste perception using an animal model. We applied NFPs to the oral cavity of mice and monitored changes in taste preference behavior and gustatory nerve activity. We found that mice treated with NFPs exhibited enhanced preferences for sweet and salty stimuli, and increased taste nerve responses to these stimuli. These results indicate that NFPs selectively enhance sweet and salty taste responses at the peripheral level and may support future translational applications aimed at improving the palatability of reduced-sugar or reduced-salt foods.

## 2. Methods

### 2.1. Compounds

The carbohydrate-based emulsion (NFPs; novel food-derived particles) was prepared by mixing palm oil (13%), α-CD (8%), and xanthan gum (0.3%) (all purchased from Fujifilm Wako Pure Chemical Corporation, Osaka, Japan) and purified water (as described in the method detailed in patent no. P7383867). As a control (Ctrl), a solution containing the same components but not formulated into an emulsion was prepared. A 10% solution of each substance was used. The 10% concentration of NFPs was used, which was selected based on preliminary evaluations indicating that this level provided adequate oral coating under test conditions. The following taste solutions were used in this study: sucrose, NaCl, citric acid, quinine hydrochloride (QHCl), ammonium chloride (NH_4_Cl) (Fujifilm Wako Pure Chemical Corporation) and L-monopotassium glutamate (MPG) (Sigma-Aldrich, St. Louis, MO, USA). All taste solutions were dissolved in distilled water (DW) [38].

### 2.2. Animals

All procedures complied with Kyushu University guidelines and were approved by the institutional animal care and use committee (approval no. A24-406-0). A total of 151 C57BL/6J mice were purchased from Kyudo (Saga, Japan). Mice were group-housed (five animals per cage) under a 12 h light/12 h dark cycle at 23 °C and had ad libitum access to water and food pellets (CE-2, CLEA, Tokyo, Japan). Mice aged 8–12 weeks were used for all experiments [38]. Animals were selected using simple randomization, in which individuals were randomly chosen from cages according to the testing order. Experimenters were not blinded to treatment conditions because the nerve recording procedures involve practical constraints, including the requirement to maintain stable neural responses. In behavioral experiments, mice that did not meet training criteria were excluded from analysis. In nerve recording experiments, animals were excluded if adequate anesthesia control or stable baseline neural responses could not be maintained. While a formal a priori power analysis was not performed, sample sizes were determined based on ranges commonly used in mouse taste behavioral and neurophysiological studies and were sufficient to detect treatment-related differences using the statistical analyses applied in this study [38,39,40,41].

### 2.3. Taste Behavior Assessment in Mice

Preference behavior was evaluated using a two-bottle choice paradigm in which mice received oral pre-exposure to the NFP or Ctrl solution before testing, with the training and deprivation schedules adapted from standard protocols and refined for this study (general procedures as in prior reports, with modifications). Training was conducted to teach the animals how to lick. Each animal was deprived of water for 23 h and housed in a licking system cage manufactured by Merquest Inc. (Toyama, Japan) on the first day of training. During a 1 h session, free access to DW was permitted. From the second to the fifth day of training, the animals were trained to drink DW for 5 s. Next, the animals were trained to select their preferred liquid in a two-bottle choice test. After 23 h of withholding water, each animal was placed in a two-bottle system cage (Merquest Inc.) for 5 min and allowed to select between DW and sucrose (150 mM) (preferred by all mice), and trained to select their preferred liquid in a two-bottle choice test over a 2-week period. Immediately before the two-bottle preference test, the animals were presented with NFP solution using the licking system during the first 5 s of the first tongue lick. The equivalence of licking among the NFP, Ctrl, and DW groups during the 5 s pre-exposure period was confirmed (Figure S1A). The 5 s exposure period was chosen because it was sufficient to distribute the solution across the oral cavity while being short enough to avoid reducing the motivation to lick during the subsequent two-bottle test. The subsequent 5 min two-bottle test was chosen because the aim of this study was to evaluate short-latency, taste-driven behavioral responses corresponding to peripheral gustatory activation. To ensure stable measurement within this short window, a pre-training protocol was implemented to reduce variability in licking behavior. After the training period, each animal was administered the solution orally, followed by presentation of two bottles: one bottle containing DW and the other containing the test solution [sucrose (60 mM), NaCl (100 mM), citric acid (10 mM), QHCl (0.1 mM), or MPG (100 mM)] for 5 min. The selection ratio of the test solution was expressed as the total intake divided by the intake of the test solution [42,43,44]. The concentrations of taste stimuli used in the behavioral tests were selected based on previous murine studies employing two-bottle assays, ensuring that all concentrations correspond to commonly used baseline levels in mouse taste behavior research [42,43,44].

### 2.4. Gustatory Nerve Recording in Mice

Recordings followed established procedures for chorda tympani (CT) whole-nerve recordings, with minor modifications for our stimulus set (general framework in refs [38,42,43,44]). Under pentobarbital anesthesia (50–60 mg/kg, i.p.), the trachea was cannulated and the head fixed in the supine position. After removing the pterygoid muscle, the right CT nerve was isolated near its entry to the bulla, transected, and placed on an Ag/AgCl recording electrode; a reference electrode was inserted into adjacent tissue. Signals were amplified (K-1; Iyodenshikagaku, Nagoya, Japan), monitored audiovisually, integrated with a 1.0 s time constant, and digitized (PowerLab/sp4; ADInstruments, Bella Vista, NSW, Australia). For anterior tongue stimulation, the fungiform field was enclosed in a silicone-rubber flow chamber. Solutions were gravity-perfused for 30 s with ~1 min DW rinse between applications. The stimulus panel comprised NH_4_Cl (100 mM), sucrose (100–1000 mM), saccharin (3–30 mM), NaCl (100–500 mM), citric acid (10–30 mM), QHCl (10–20 mM), and MPG (100–300 mM). After baseline series acquisition, NFP or Ctrl was applied to the tongue, and the series was repeated. Only stable recordings were analyzed. Stable recordings were defined as those in which baseline activity and the response to 100 mM NH_4_Cl did not show qualitative drift during the session. Each stimulus was applied 2–3 times. Rinsing was performed under standardized conditions (~1 min DW), but when the response failed to fully return to baseline, the rinse duration was extended. The next stimulus was delivered only after complete recovery to baseline had been confirmed. These concentration ranges are consistent with previous CT recording studies in mice [38,39,40,41], allowing comparison with established dose–response characteristics. Response magnitude was computed from the integrated trace as the mean over the central 20 s of each 30 s stimulus (excluding the first and last 5 s) and normalized to the response to 100 mM NH_4_Cl to reduce inter-animal variability.

### 2.5. Statistical Analysis

**Figure 1:**
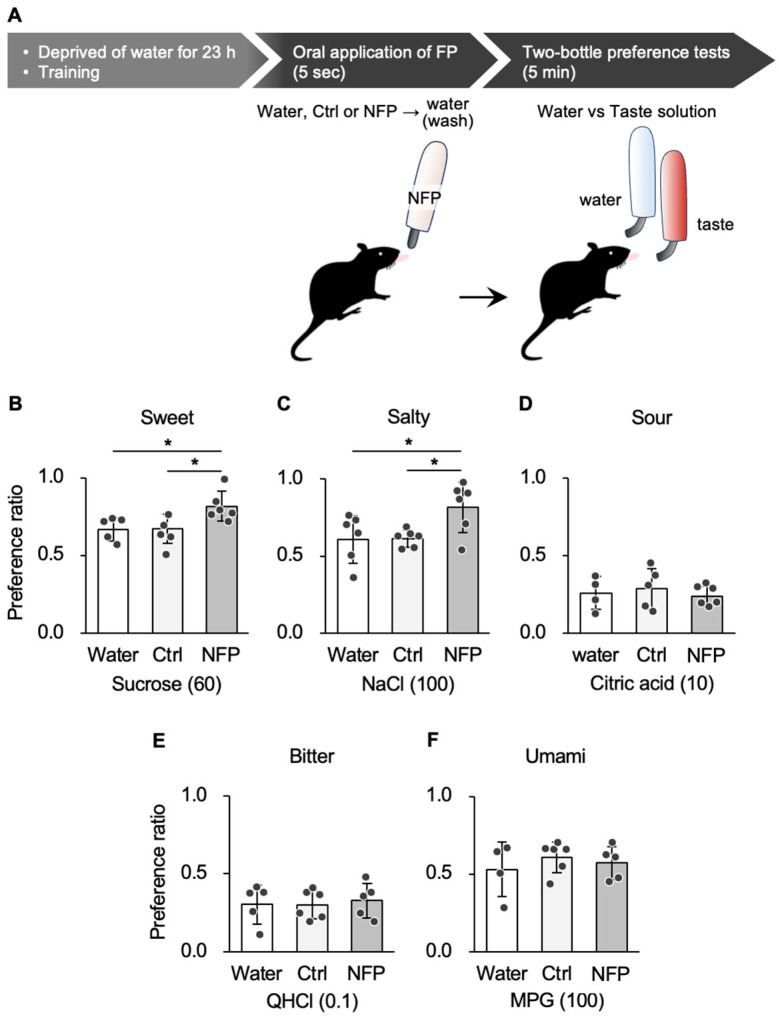
Oral application of NFPs enhances sweet and salty taste preferences in mice. (**A**) Experimental schedule, (**B**–**F**), mean preference ratios for taste substances during 5 min of consumption in two-bottle preference tests after oral application of water (white), Ctrl (light gray) and NFPs (dark gray). Taste stimuli were 60 mM sucrose (**B**), 100 mM NaCl (**C**), 10 mM citric acid (**D**), 0.1 mM QHCl (**E**), and 100 mM monopotassium glutamate (MPG) (**F**). Preference ratio was calculated as the intake of the taste solution divided by the total intake. Sample sizes were *n* = 4–6 for the water group, *n* = 5–6 for the Ctrl group, and *n* = 5–6 for the NFP group. NFP application significantly increased preference ratios for sucrose and NaCl compared with both the water and Ctrl groups. Values are mean ± SD. Asterisks indicate statistically significant differences (* *p* < 0.05, one-way ANOVA followed by Tukey’s post hoc test). Black dots represent individual animals.

For behavioral taste preference tests, when only two groups were compared (e.g., Figure S1B), data were analyzed by unpaired *t*-test. When three or more groups were compared (e.g., Figure 1), data were subjected to one-way analysis of variance (ANOVA), followed by Tukey’s post hoc test for multiple comparisons if the ANOVA was significant. For CT nerve responses, data were analyzed using two-way ANOVA with factors for treatment (NFP vs. Ctrl) and concentration, followed by Bonferroni-corrected unpaired *t*-tests when appropriate. All analyses were conducted using IBM SPSS Statistics 26.0 (IBM Corp., Armonk, NY, USA).

## 3. Results

### 3.1. Effect of NFPs on Taste Preference Behavior in Response to Taste Stimuli

First, to investigate the effects of oral application of NFPs on preference behavior in mice, two-bottle choice tests were conducted. Prior to each test, mice were administered either with the Ctrl solution (the same components as NFPs but not in an emulsion form) or NFPs. These substances were applied to the oral cavity by licking a set amount for 5 s (Figure S1A). A two-bottle preference test was then conducted, with one bottle containing water and the other containing either Ctrl or NFPs, for 5 min. The results showed that the mice did not show a preference for Ctrl or NFPs (Figure S1B).

Mice that received NFPs showed significantly higher preference ratios for both sucrose (sweet) and NaCl (salty) compared with the group that received water (one-way ANOVA and Tukey’s post hoc, *p* < 0.05; effect of application; Figure 1A,B; Table 1). However, no significant changes in preference for citric acid (sour), QHCl (bitter), or MPG (umami) were observed (one-way ANOVA, *p* > 0.05; effect of application; Figure 1C–E; Table 1). These findings indicate that the effect of NFPs is selective to sweet and salty stimuli. Additionally, compared with the Ctrl group, the mice that received NFPs showed significantly higher preference ratios for both sucrose and NaCl (one-way ANOVA and Tukey’s post hoc, *p* < 0.05; effect of application; Figure 1A,B; Table 1). No significant changes in preference for the other tastes were observed (one-way ANOVA, *p* > 0.05; effect of application; Figure 1C–E; Table 1). Because NFPs and Ctrl contain the same components, these results suggest that the enhancement of sweet and salty preference requires the emulsion (Pickering particle) structure of NFPs rather than the components themselves.

### 3.2. Effect of NFPs on Gustatory Nerve Responses to Taste Stimuli

**Figure 2:**
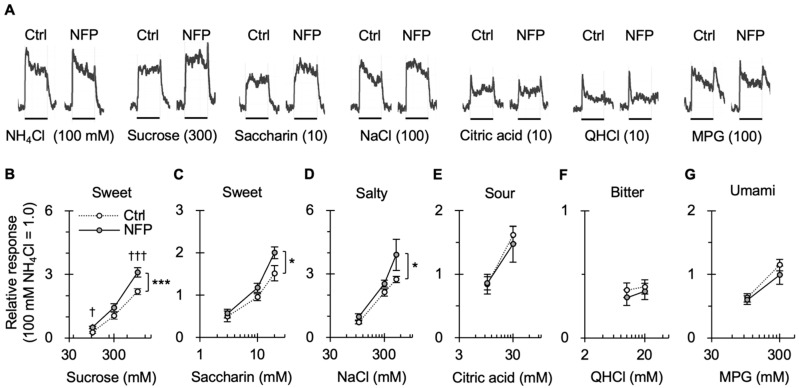
NFP application to the tongue enhances CT nerve responses to sweet and salty stimuli in mice. (**A**) Typical examples of CT nerve responses to 100 mM NH_4_Cl (NH_4_Cl), 300 mM sucrose, 10 mM saccharin, 100 mM NaCl, 10 mM citric acid, 10 mM quinine-HCl (QHCl), and 100 mM MPG, in Ctrl and NFP-applied mice. Bars indicate taste stimulation (30 s). (**B**–**G**), Concentration response relationships of CT nerve responses for various taste substances in Ctrl and NFP-applied mice. Taste stimuli were 100–1000 mM sucrose (**B**), 3–30 mM saccharin (**C**), 100–500 mM NaCl (**D**), 10–30 mM citric acid (**E**), 10–20 mM QHCl (**F**), and 100–300 mM MPG (**G**). CT nerve responses in Ctrl and NFP-applied mice (*n* = 6–8 for Ctrl, *n* = 6–7 for NFP) were normalized to the response to 100 mM NH_4_Cl. Values are mean ± SEM, * *p* < 0.05, *** *p* < 0.001, two-way ANOVA, effect of NFP. † *p* < 0.02, ††† *p* < 0.003 (unpaired *t*-test with Bonferroni correction). To clarify statistical interpretation, asterisks denote significant main effects of treatment (NFP vs. Ctrl) in two-way ANOVA, and daggers indicate significant differences at individual concentrations based on Bonferroni-corrected unpaired *t*-tests.

To investigate whether NFPs influence the peripheral taste response, we recorded the taste responses to various taste stimuli from the CT nerve, innervating the anterior part of the tongue after applying NFPs or Ctrl to the tongue. Consistent with the behavioral results, NFP application significantly enhanced CT responses to sucrose, saccharin (sweet), and NaCl (salty), compared with those of mice in the Ctrl group (two-way ANOVA, *p* < 0.05; Figure 2A–D; Table 1). In contrast, the responses to sour, bitter, and umami solutions were not significantly different between groups (two-way ANOVA, *p* > 0.05; Figure 2E–G; Table 1). These results demonstrate that NFPs enhance both the behavioral preference and peripheral gustatory nerve responsiveness to sweet and salty stimuli. Because this enhancement was not observed for Ctrl, the findings suggest that the emulsion-based physicochemical structure of NFPs directly modulates taste signal transduction at the level of peripheral taste organs.

In this study, we demonstrated that novel food-derived particles, NFPs, selectively enhance taste behavior and taste nerve responses to sweet and salty stimuli in mice. Oral administration of NFPs significantly increased preference rates for sweet (sucrose) and salty (NaCl) flavors, and enhanced taste nerve responses to sweet (sucrose, saccharin) and salty stimuli. The enhanced sweet and salty taste responses observed in both short-term taste behavior tests and gustatory nerve recordings suggest that NFPs influence peripheral taste sensing. These behavioral and neural results together indicate that NFPs modulate peripheral taste sensitivity rather than motivational or novelty-related processes.

#### 3.2.1. Mechanistic Considerations

Because the present study did not directly examine physicochemical or molecular mechanisms, the following explanations should be regarded as hypotheses derived from prior literature. One possibility is that the Pickering emulsion structure of NFPs transiently forms a hydrophilic, xanthan-rich layer on the oral epithelium. Xanthan gum is a viscous and mucoadhesive polysaccharide known to adhere to oral surfaces [45,46,47,48].

**Figure 3:**
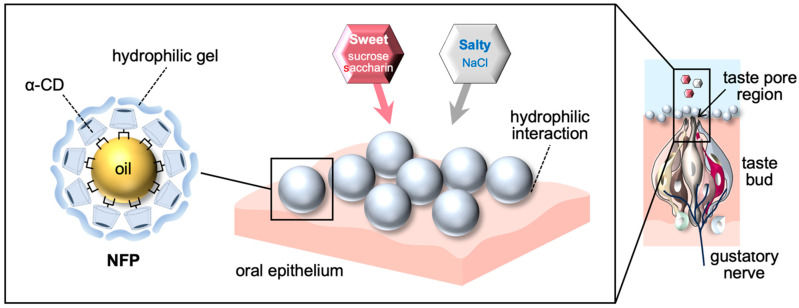
Proposed hypothetical mechanism of NFP-mediated taste enhancement.

The physicochemical properties of this putative layer may also influence its interactions with different taste substances as illustrated in Figure 3. Acidic solutions such as citric acid, QHCl, and MPG can protonate xanthan gum and reduce its solubility [49], potentially diminishing retention. In contrast, neutral sweeteners such as sucrose and saccharin may be retained more readily through hydrogen-bonding interactions with hydroxyl or imide groups on the emulsion surface [50,51]. These mechanisms remain speculative but may partly explain the selective enhancement of sweet and salty taste responses observed in this study. Definitive confirmation will require biochemical, biophysical, and rheological analyses.

#### 3.2.2. Implications for Food Matrix-Based Modulation

The mice showed no inherent preference for NFPs itself (Figure S1B), indicating that the enhancement of sweet and salty taste responses is not due to the palatability of NFPs but instead reflects its interaction with taste stimuli. Unlike receptor-active taste enhancers such as kokumi γ-glutamyl peptides or natural high-potency sweeteners (e.g., steviol glycosides, mogrosides), NFPs modulate taste responses without introducing taste-active compounds and instead relies on matrix-derived physicochemical properties. A novel finding of this study is that the structural characteristics of the food matrix itself can selectively influence peripheral taste responses, independent of added flavor molecules. Given that α-CD and xanthan gum are widely used in food applications, the NFP platform may provide a practical strategy for enhancing sweetness or saltiness in reduced-sugar and reduced-salt products without introducing additional taste-active compounds. Reducing sugar and salt intake is an important strategy for preventing lifestyle-related diseases such as obesity, kidney disease, and cardiovascular disease [4,5,6,7]. However, consumers frequently report reduced-sugar and reduced-salt foods as being less palatable [12]. Our findings therefore suggest a potential approach for improving the acceptability of healthier food formulations.

#### 3.2.3. Limitations

This study has several limitations that should be considered when interpreting the findings. The two-bottle preference test can be influenced by motivational and novelty-related factors, although extended training helped minimize these effects. In addition, only one concentration and exposure duration of NFPs were tested, and therefore dose–response and retention-time effects remain unknown. Sample sizes, particularly for CT recordings, were modest but remained within the standard range used in mouse taste studies. The variability observed in sour, bitter, and umami responses is not unexpected in taste research but should be interpreted with appropriate caution. Moreover, no physicochemical characterization of NFPs (e.g., particle size, viscosity, or interfacial behavior under saliva-like conditions) was performed, and thus mechanistic interpretations remain hypothetical. Finally, because this work was conducted in mice, the translational relevance of NFPs will require confirmation in human sensory and toxicological studies.

#### 3.2.4. Future Directions

Future research should include systematic analysis of NFP physicochemical properties, dose–response relationships, residence time on oral surfaces, and interactions with sweet and salty compounds. Human sensory evaluation, consumer acceptability testing, and comprehensive safety assessment—including toxicological evaluation within relevant food-safety regulatory frameworks—will be essential for establishing applicability of NFPs to reduced-sugar or reduced-salt food products.

## 4. Conclusions

In conclusion, this study demonstrates that food matrix-based modulation using NFPs enhances peripheral sweet and salty taste responses. Although mechanistic explanations remain hypothetical, NFPs may represent a practical strategy for improving palatability in reduced-sugar and reduced-salt foods. Further characterization and human validation will clarify the potential of NFPs as an innovative tool for supporting healthier dietary practices.

## Figures and Tables

**Table 1:** Results of statistical analysis.

Fig.	Content		Analysis			*p* Value
S1A	Water vs. Ctrl, Water vs. NFP, Ctrl vs. NFP	*n* = 9 (Water), 9 (Ctrl), 9 (NFP)	One-way ANOVA	F (2, 26) = 0.094		0.911
S1B	Application (Ctrl vs. NFP)	*n* = 7 (Ctrl), 7 (NFP)	Unpaired *t*-test	Ctrl vs. NFP		0.465
1B	Sucrose 60 mM (Water vs. Ctrl, Water vs. NFP, Ctrl vs. NFP)	*n* = 4–6 (water), 5–6 (Ctrl), 5–6 (NFP)	One-way ANOVA	F (2, 15) = 6.501		0.011
			Post hoc Tukey’s test	Water vs. Ctrl Water vs. NFP Ctrl vs. NFP		0.845 0.041 0.014
1C	NaCl 100 mM (Water vs. Ctrl, Water vs. NFP, Ctrl vs. NFP)		One-way ANOVA	F (2, 17) = 4.748		0.025
			Post hoc Tukey’s test	Water vs. Ctrl Water vs. NFP Ctrl vs. NFP		0.996 0.040 0.047
1D	CA 10 mM (Water vs. Ctrl, Water vs. NFP, Ctrl vs. NFP)		One-way ANOVA	F (2, 14) = 0.301		0.745
1E	QHCl 0.1 mM (Water vs. Ctrl, Water vs. NFP, Ctrl vs. NFP)		One-way ANOVA	F (2, 15) = 0.091		0.914
1F	MPG 100 mM (Water vs. Ctrl, Water vs. NFP, Ctrl vs. NFP)		One-way ANOVA	F (2, 14) = 0.484		0.628
2B	Injection (Ctrl vs. NFP) × concentration [sucrose]	*n* = 6–8 (Ctrl), 6–7 (NFP)	Two-way ANOVA	Injection (Ctrl vs. NFP) Concentration Injection × concentration	F (1, 39) = 17.503 F (2, 39) = 119.726 F (2, 39) = 2.890	<0.001 <0.001 0.068
			Unpaired *t*-test with Bonferroni correction	Ctrl vs. NFP	100 mM sucrose 300 mM sucrose 1000 mM sucrose	0.004 0.049 0.001
2C	Injection (Ctrl vs. NFP) × concentration [saccharin]		Two-way ANOVA	Injection (Ctrl vs. NFP) Concentration Injection × concentration	F (1, 39) = 5.984 F (2, 39) = 46.078 F (2, 39) = 1.369	0.019 <0.001 0.266
			Unpaired *t*-test with Bonferroni correction	Ctrl vs NFP	3 mM saccharin 10 mM saccharin 30 mM saccharin	0.232 0.041 0.019
2D	Injection (Ctrl vs. NFP) × concentration [NaCl]		Two-way ANOVA	Injection (Ctrl vs. NFP) Concentration Injection × concentration	F (1, 33) = 5.880 F (2, 33) = 32.584 F (2, 33) = 1.223	0.021 <0.001 0.307
			Unpaired *t*-test with Bonferroni correction	Ctrl vs NFP	100 mM NaCl 300 mM NaCl 500 mM NaCl	0.040 0.041 0.041
2E	Injection (Ctrl vs. NFP) × concentration [citric acid]		Two-way ANOVA	Injection (Ctrl vs. NFP) Concentration Injection × concentration	F (1, 22) = 0.109 F (1, 22) = 13.436 F (1, 22) = 0.156	0.744 0.001 0.696
2F	Injection (Ctrl vs. NFP) × concentration [QHCl]		Two-way ANOVA	Injection (Ctrl vs. NFP) Concentration Injection × concentration	F (1, 20) = 0.606 F (1, 20) = 0.374 F (1, 20) = 0.038	0.445 0.548 0.848
2G	Injection (Ctrl vs. NFP) × concentration [MPG]		Two-way ANOVA	Injection (Ctrl vs. NFP) Concentration Injection × concentration	F (1, 22) = 0.915 F (1, 22) = 20.611 F (1, 22) = 0.400	0.349 <0.001 0.534

## Data Availability

The data that support the findings of this study are available from the corresponding authors, upon reasonable request.

## Supplementary Materials

The following supporting information can be downloaded at: https://www.mdpi.com/article/10.3390/nu18010098/s1, Figure S1: Preference tests after oral application of NFP in mice. *A.* Comparison of oral application of water (white), Ctrl (light gray), and NFP (dark gray) by 5 s of licking. ***B***, Preference for Ctrl and NFP itself. In a 5-min two-bottle preference test, average preference ratios were measured when water vs. Ctrl and water vs. NFP were presented, and the groups were compared. Values are mean ± SD. NFP, novel food-derived particles consisting of lipid, α-cyclodextrin, and xanthan gum; Ctrl, same components as NFP but not in emulsion form.
