# Supplementary material for: A Novel Food-Derived Particle Enhances Sweet and Salty Taste Responses in Mice
Source: Nutrients. 2025 Dec 27;18(1):98. doi: 10.3390/nu18010098 (PMC12787338; doi:10.3390/nu18010098)
Supplement: Supplementary file 1 [file nutrients-18-00098-s001.zip › nutrients-4009454-supplementary.pdf]

Figure S1

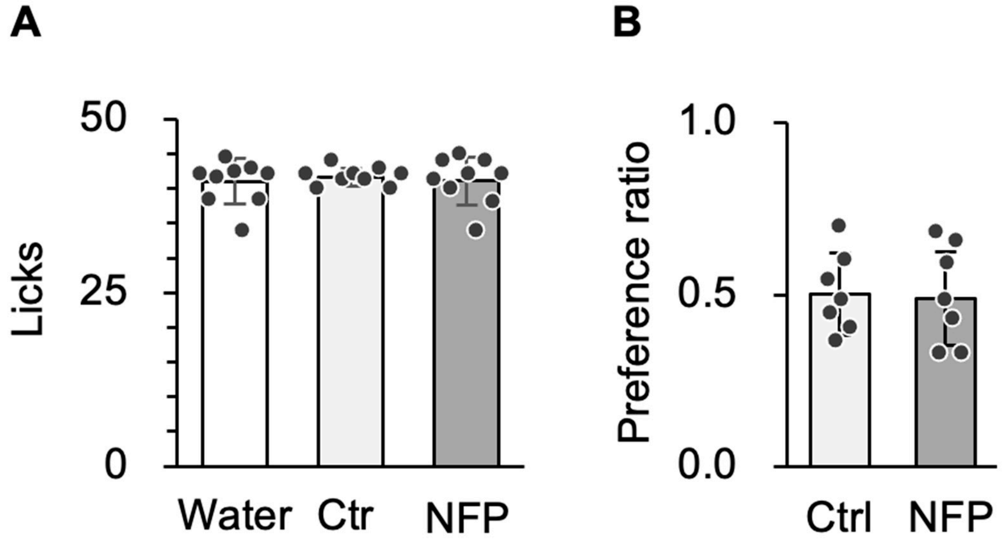

Figure S1. Preference tests after oral application of NFP in mice. ***A.*** Comparison of oral application of water (white), Ctrl (light gray), and NFP (dark gray) by 5 seconds of licking. ***B***, Preference for Ctrl and NFP itself. In a 5-minute two-bottle preference test, average preference ratios were measured when water vs. Ctrl and water vs. NFP were presented, and the groups were compared. Values are mean ± SD. NFP, novel food-derived particles consisting of **lipid, α-cyclodextrin, and xanthan gum**; Ctrl, same components as NFP but not in emulsion form.
